# Editorial: Neural mechanisms of cognitive control and emotion in birds

**DOI:** 10.3389/fphys.2022.1028103

**Published:** 2022-09-29

**Authors:** András Csillag, Toshiya Matsushima

**Affiliations:** ^1^ Department of Anatomy, Histology and Embryology, Budapest, Hungary; ^2^ Department of Biological Sciences, Graduate School of Science, Hokkaido University, Sapporo, Japan

**Keywords:** avian brain, learning, lateralization, reward, songbird

In the present Research Topic, we have been successful in attracting contributions from a wide range of research centers around the globe. The new results gathered in a variety of biological disciplines represent important advances toward a better understanding of emotion and cognitive control in birds. Out of 21 articles by 67 authors (2 brief original research reports, 15 original research reports, and four review articles including one systematic review study), 10 studies focused on galliform precocial birds (and their hatchlings), nine on passerine birds (songbirds including crows and Java sparrows), and two on pigeons. By reviewing these studies, we hope to provide an update on cutting edge results hallmarking knowledge on avian brain and behaviors of today. We are grateful for the contributors, the guest editors and the reviewers for their efforts and intriguing ideas, which, so we believe, will set the foundation for future progress in avian physiology in a highly comprehensive manner, ranging across the fields of behavioral, anatomical, endocrinological, biochemical and evolutionary biology.

## Stress, emotion, and intrinsic reward system for singing

Animals are under continuous and endless barrages of stressors both from ambient physical world and social life. Kato et al. reports their novel finding on the molecular basis of stress responses and specified neurosecretory protein GL/GM (NPGL/NPGM), which acts as the stress mediator in the mediobasal hypothalamus. In particular, social isolation stress causes an acute increase in the expression of these proteins. Pross et al. examined the extended amygdala (division of the dorsal striatum) and found multiple types of enkephalin cells involved in stress regulation. Detailed neurochemical identification of the limbic network will constitute the critical basis for physiological specification of stress responses. Comparisons of songbirds (zebra finches ZFs and Bengalese finches BFs) led Kim et al. to suggest that spontaneous singing (undirected songs, United States) could be associated with environmental stress. These two species of finches revealed striking differences in the frequency of “dark singing,” where BFs emit United States much more than ZFs. Ambient light condition critically controls the singing motivation, but with a strong species difference.

Rich lines of evidence and ideas seem to converge on the positive emotional aspects of singing. Singh and Iyenga reviewed the opioid receptors in songbird brain and suggested that birds regulate singing and song learning by intrinsic motivational control. In accordance, the review by Riters et al. suggests that the nucleus accumbens in the medial striatum could be the site for intrinsic reward system that enables birds to proactively seek for mate. Kim and Kojima in their second paper, presented endocannabinoids as the activators of undirected spontaneous singing in songbirds. Study of singing behavior does not only tackle the issues of learned vocalization, but also those of its emotional/motivational background.

### Lateralization and its evolutionary background

Lateralized hemispheric control is widely observed in sensory perception, cognition, and executive control of behaviors. Protti-Sánchez et al. reports a unique case where the umami-tastes (tasting of amino acids) yield lateralized activation in the chick limbic nucleus taeniae. Xiao and Güntürkün further expand the scope toward an associative cortex, or “prefrontal”-like avian analogue, the nidopallium caudolaterale (NCL) of pigeons. They stress the importance of sequential build-up of lateralization through the cascade of signal processing in the visual system. The detailed study on transmitter receptors in NCL by Herold et al. should also be referred to in the light of functional lateralization. Readers of the subject often feel lost in the plethora of literature, unable to construct the framework for understanding these issues. The systematic review article by Soma provides us with such a framework, i.e., a comprehensive survey of visual lateralization. In particular, the finding of opposite lateralization patterns in passerine and non-passerine birds strikingly underlines the importance of evolutionary perspectives.

### Cellular and molecular substrates of learning

Studies of filial imprinting are still actively progressing. Aoki et al. propose a novel and quantitative method of imprinting that allows partial constraint of the body and head in awake chicks. Meparishvili et al. pointed out tyrosine kinase (Src) as a novel key player in innate predisposition and engram formation in the pallial network of domestic chicks. It is also noted that the kinase system plays a critical role in execution of social behaviors of adults, just as Moaraf et al. revealed the involvement of GSK-3β in the social behaviors of zebra finches; its inhibition caused birds to move closer to a stranger than to a familiar companion, furthermore, the treated birds showed hyperactivity. The cellular target of the thyroid hormone, another player critically involved in the sensitive period, was examined by Saheki et al., who revealed that the hormone acutely enhances GABA-AR mediated synaptic inhibition, supporting the idea that the excitation-inhibition balance is critical for the control of critical period.

### Structure and molecular architecture of the avian brain

Beside function, morphology of the avian brain is also subject to developmental control. Stingo-Hirmas et al. give us a unique example where the size of cerebellum could be a predictor of the habituation to fearful objects in adult chickens. Also, the importance of careful behavioral testing and control of genetic background are being stressed in this study. One should not overlook the importance of descriptive studies such as detailed information on gene expression either. The avian hippocampus has been assumed to lack distinct subdivisions and layer structures. Fujita et al. present a significant contribution to our understanding of the avian hippocampus by detailed *in situ* hybridization analysis of serotonin receptor subtypes. In another paper, Fujita et al. were successful in the morphological dissociation between the dorsal and medial raphe in the chick brainstem by the same set of serotonin receptor subfamilies. Acute behavioral pharmacology in quails by Pichová et al. clearly showed functional control of reward expectation by the dopamine system. Notably, the manipulation of D1 and D2 receptors by antagonists yielded coherent rather than opposite results, meaning that both receptor types are likely involved in the given aspects of reward prediction.

### Comprehensive studies for prospect and future steps

Several new and powerful techniques have been applied to unravel the confounding issues of emotional control. Pendergraft et al. applied FDG-PET imaging of the brain in awake crows, and revealed that the activation of the nucleus taeniae amygdala (limbic) and nidopallium caudolaterale (NCL, medial portion) requires multimodal combination of multimodal stimuli of conspecifics, namely the sight of food and the sound of conspecific vocalization during foraging. Similarly innovative approach was successfully applied in the study of event-related mismatch responses reported in Java sparrows by Mori and Okanoya. Field potentials recorded from the auditory area of telencephalon (NCM) revealed significant effects of deviations from the repeated/habituated sounds in both pure tone and natural vocalization. Furthermore, the same team Fujii et al. contributes a comprehensive review on the question of why females are so choosy about male songs. As with all other biological disciplines, physiological mechanisms should be studied in the light of evolution/adaptive values, because evolutional thinking is a rich source of novel approaches in physiology.

**Table of contributions:** 

title	first author	species	methods	main conclusion
Effect of Stressors on the mRNA Expressions of Neurosecretory Protein GL and Neurosecretory Protein GM in Chicks	Masaki Kato	chicks	gene expression of regulatory proteins	NPGM, but not NPGL or HDC, participates in several physiological responses to stress in chicks.
Developmental-Based Classification of Enkephalin and Somatostatin Containing Neurons of the Chicken Central Extended Amygdala	Alessandra Pross	chicken (adults)	gene expression of neuromodulators	Enkephalin cells likely derive from the dorsal striatal division (extended amygdala) as the basis for studying the role of these cells in stress regulation.
Effect of Darkness on Intrinsic Motivation for Undirected Singing in Bengalese Finch (Lonchura striata Domestica): A Comparative Study with Zebra Finch (Taeniopygia guttata)	Yunbok Kim	songbirds	behavioral study and blood corticosterone	Differences between two species of finch provide insights into the interactions among singing motivation, ambient light, and environmental stress.
The Role of the Endogenous Opioid System in the Vocal Behavior of Songbirds and Its Possible Role in Vocal Learning	Utkarsha A. Singh	songbirds	gene expression of transmitter receptors	Expression pattern of opioid receptors and vocal learning in songbird.
Birdsong and the Neural Regulation of Positive Emotion	Lauren V. Riters	songbirds	behavioral analysis and neuroanatomy	Nucleus accumbens contributes to positive emotional states that motivate and reward singing behavior and the responses to song.
Contribution of Endocannabinoids to Intrinsic Motivation for Undirected Singing in Adult Zebra Finches	Yunbok Kim	songbirds	behavioral pharmacology	Endocannabinoids are critically involved in the regulation of intrinsic motivation of undirected singing.
Activation of the Nucleus Taeniae of the Amygdala by Umami Taste in Domestic Chicks (Gallus gallus)	Francesca Protti-Sánchez	chicks	c-Fos imaging	Neuronal responses to umami and bitter tastes are lateralized in nucleus taeniae of the amygdala, a region processing reward information.
“Prefrontal” Neuronal Foundations of Visual Asymmetries in Pigeons	Qian Xiao	pigeons	*in vivo* single unit electrophysiology	The hemispheric asymmetries for visual discrimination are realized by a sequential buildup of lateralized neuronal responses in the avian forebrain.
Behavioral Training Related Neurotransmitter Receptor Expression Dynamics in the Nidopallium Caudolaterale and the Hippocampal Formation of Pigeons	Christina Herold	pigeons	gene expression of transmitter receptors	Patterns of neurotransmitter receptor expression in the avian prefrontal cortex and the hippocampal formation suggest association to learning and memory.
Behavioral and Evolutionary Perspectives on Visual Lateralization in Mating Birds: A Short Systematic Review	Masayo Soma	passerine and non-passerine birds	phylogenetic comparative methods	Passerine and non-passerine species showed opposite eye use for mating, which could have stemmed from a difference in altricial *vs.* precocial development.
Imprintability of Newly Hatched Domestic Chicks on an Artificial Object: A Novel High Time-Resolution Apparatus Based on a Running Disc	Naoya Aoki	chicks	behavioral study of imprinting	New apparatus was developed to detect behavioral changes during imprinting.
Src and Memory: A Study of Filial Imprinting and Predispositions in the Domestic Chick	Maia Meparishvili	chicks	biochemistry of learning	One pool of Src (tyrosine kinase) reflects the chick's predisposition to learn, while the second pool the inhibited condition as a result of learning.
GSK-3β Inhibition in Birds Affects Social Behavior and Increases Motor Activity	Stan Moaraf	zebra finch	behavioral pharmacology	Inhibition of GSK-3β acutely affected the social behavior and caused hyperactivity.
Suppressive Modulation of the Chick Forebrain Network for Imprinting by Thyroid Hormone: An *in Vitro* Study	Yuriko Saheki	chicks	slice electrophysiology, pharmacology	Thyroid hormone enhanced GABA-A action and suppressed the NMDA-R in IMM neurons, but the synaptic potentiation remained unchanged.
Proportional Cerebellum Size Predicts Fear Habituation in Chickens	Diego Stingo-Hirmas	chicken (adults)	neuroanatomy	Proportional cerebellum size does not predict an individual’s fear response, but rather the habituation process to a fearful stimulus.
Chick Hippocampal Formation Displays Subdivision- and Layer-Selective Expression Patterns of Serotonin Receptor Subfamily Genes	Toshiyuki Fujita	chicks	*in situ* hybridization of receptor subtypes	Subfamilies of the serotonin receptor genes show subdivision- and layer-selective expression patterns in the hippocampus.
Serotonergic Neurons in the Chick Brainstem Express Various Serotonin Receptor Subfamily Genes	Toshiyuki Fujita	chicks	*in situ* hybridization of receptor subtypes	The expression pattern of 5-HT receptors in the serotonin neurons of chick DR and MR may vary, suggesting heterogeneity among and within the serotonin neurons of the DR and MR in the chick brainstem
The Acute Pharmacological Manipulation of Dopamine Receptors Modulates Judgment Bias in Japanese Quail	Katarína Pichová	Japanese quail	behavioral pharmacology	Dopamine D1 and D2 receptor blockade leads to a decrease in the reward expectation and the negative judgment of stimuli.
American Crow Brain Activity in Response to Conspecific Vocalizations Changes When Food Is Present	Loma John T. Pendergraft	crows	FDG-PET imaging	The nucleus taenia (TnA) and caudal nidopallium show increased activity in response to conspecific calls if audio/visual stimuli are combined; a PET imaging study.
Mismatch Responses Evoked by Sound Pattern Violation in the Songbird Forebrain Suggest Common Auditory Processing with Human	Chihiro Mori	Java sparrow	event-related potential (ERP)	Violation of the triplet sequence pattern elicits mismatch negativity, indicating the ability to extract sound sequence in the auditory forebrain.
Song Preference in Female and Juvenile Songbirds: Proximate and Ultimate Questions	Tomoko G. Fujii	songbirds	Tinbergen's 4 questions	The current understanding of song preference in female and juvenile songbirds are summarized in the context of Tinbergen’s four questions.

